# Tranexamic acid and reduction of blood transfusion in lower limb trauma surgery: a randomized controlled study

**DOI:** 10.1051/sicotj/2021053

**Published:** 2021-10-28

**Authors:** Gurleen Kaur, Harpal Singh Selhi, Naresh Jyoti Delmotra, Jaspreet Singh

**Affiliations:** 1 Professor, Department of Pharmacology, Adesh Medical College & Hospital Kurukshetra Haryana 136135 India; 2 Professor, Department of Orthopaedics, Dayanand Medical College & Hospital Ludhiana Punjab 141001 India; 3 Professor & Head, Department of Pharmacology, Adesh Medical College & Hospital Kurukshetra Haryana 136135 India; 4 Assistant Professor, Department of Orthopaedics, Government Medical College Patiala Punjab 147001 India

**Keywords:** Tranexamic acid, Deep Vein Thrombosis, Blood transfusion

## Abstract

*Introduction*: Post-operative blood loss in lower limb trauma fractures increases morbidity. Very few studies have evaluated the efficacy of Tranexamic Acid (TXA) in reducing blood loss and the consequent requirement of blood transfusion in the Indian population. *Methods*: This was a randomized controlled study of 100 patients with lower limb trauma. Fifty patients were given 1 g of TXA before surgery, and 50 patients were not given TXA. The requirement of blood transfusion, fall in Hb, the number of days admitted in the hospital after surgery were recorded, and evidence of deep vein thrombosis (DVT) was monitored. *Results*: Baseline demographics between the groups were comparable. The required blood transfusion and fall in Hb in patients receiving intra-operative TXA were significantly lower than those not given TXA (*p* < 0.0001). There was no significant difference in the length of hospital stay between the two groups (*p* = 0.6). There was no significant difference in the incidence of DVT in both groups. *Discussion*: TXA helps reduce the morbidity of trauma patients by reducing the requirement for blood transfusion. Its use is safe in lower limb trauma surgery and lowers the cost of therapy to the patient.

## Introduction

Lower limb fractures account for approximately one-third of all fractures and may result in substantial mortality and morbidity [1]. Post-operative blood loss leading to anemia, produces significant morbidity like delayed mobilization. It also has been seen to increase the length of hospital stay [2, 3]. Anemia impedes functional mobility and is an independent risk factor for patients not walking post-operatively [3]. Post-operative blood loss is corrected by blood transfusion, which has its own set of complications and increases the total cost of treatment [4].

Anti-fibrinolytic agents like tranexamic acid (TXA) are used to prevent blood loss. TXA is a synthetic lysine derivative that exerts its effect by reversibly blocking lysine binding sites on plasminogen, preventing fibrin degradation [5]. While preventing blood loss, these agents are known to cause complications, like deep vein thrombosis (DVT), thrombus formation, leading to myocardial or cerebrovascular infarction, renal artery occlusion, etc. [5, 6].

The efficacy and safety of TXA in joint replacement and hip fracture surgery [7–9] have been often validated. However, the administration of TXA in trauma patients is controversial, with strong opinions for [10, 11] and against [12, 13] its use. Moreover, there is limited literature documenting its use in the Indian population. This study was done to address this deficiency and to look at the role of TXA in patients with lower limb trauma. The study aimed to record the requirement of post-operative blood transfusion, average length of hospital stay, incidence of deep vein thrombosis and other thrombotic events.

It was hypothesized that TXA use in trauma patients would reduce blood transfusion requirement without any significant increase in complications.

## Materials and methods

### Study design

This study was a single-center, prospective randomized controlled parallel-group study with 1:1 allocation. After obtaining ethical approval from IEC (Ref. No. PHMA/GSMCH-17/IEC-29A), 116 patients of lower limb trauma presenting to the Emergency Department of Civil Hospital, Rajpura between April 2017 to August 2018 were taken up for the study. Written informed consent was obtained from all the participating patients.

### Patient selection

The patients aged 18–60 years, suffering from lower limb trauma were taken up for the study. The presence of an old implant or infection at the fracture site or any blood coagulation disorder were the exclusion criteria for the study. The patients undergoing percutaneous wire placement procedures for fracture fixation were also excluded. The other exclusion criteria were polytrauma, non-consenting patients, a psychiatric patient. Sixteen patients were excluded due to old fracture, percutaneous wire fixation, or non-consenting patient. A total of 100 patients met the inclusion criteria and were enrolled.

### Pre-operative evaluation

The enrolled patients were assessed pre-operatively, and all relevant investigations are done. The patients were taken up for surgery after stabilization of the general condition. Pre-operative blood transfusion was done as required to have an Hb level of at least 9.5 mg/dL. There were 14 patients in group A and 14 patients in group B who were given blood pre-operatively. Since the number of patients receiving blood transfusion pre-operatively is the same, the difference was not significant.

### Surgical intervention

The patients were administered spinal anesthesia or general anesthesia, depending on the anesthetist’s discretion. The patients were treated by internal fixation of fracture using plates or interlocking nails. They were given antibiotic prophylaxis at the beginning of surgery. Intra-operatively, all the patients were given Ringer Lactate and intravenous colloid solution (Haemaccel) for volume replacement.

The patients enrolled were randomly divided into two groups. The random allocation of the patients to either group was done by one of the authors using a random number table. Group A had 50 patients who were given 1 g of TXA intravenously pre-operatively. IV TXA was administered, at the time of the start of the surgical incision, by mixing 1 g of TXA in 100 mL of normal saline. Group B had 50 patients who were kept as a control group and were not given TXA.

### Post-operative evaluation

The requirement of post-operative blood transfusion was recorded as a primary outcome measure. The post-operative blood transfusion was done if the patients’ Hb level fell below 9.5 mg/dL. The secondary outcome measures included fall in Hb, total hospital stay and DVT evidence. Patients of both groups were monitored by checking Haemoglobin (Hb) levels as required. The Hb readings were expressed as mean ± standard deviation and range.

Other complications were monitored daily by looking for leg swelling for DVT, breathlessness, and fall in saturation for pulmonary embolism, or a cardiac event like angina or myocardial infarction, deterioration of mental status for stroke event. For all the patients who had any of these complaints, a medical opinion was sought and managed accordingly.

The probability of venous thromboembolism was suspected by Wells et al. [14] Clinical model (Table 1) to assess the pre-test probability of DVT. Patients with a high probability were administered the D-Dimer test and other relevant tests as needed to confirm thromboembolism.

**Table 1:** Wells et al. clinical model to assess pre-test probability of deep vein thrombosis.

Clinical features	Score
Active cancer (treatment ongoing or within previous 6 months or palliative)	1
Paralysis, paresis, or recent plaster immobilization of the legs	1
Recently bedridden for more than 3 days or major surgery within 4 weeks	1
Localized tenderness along the distribution of the deep venous system	1
Entire leg swollen	1
Calf swelling by more than 3 cm compared with the asymptomatic leg (measured 10 cm below the tibial tuberosity)	1
Pitting oedema (greater in the symptomatic leg)	1
Collateral superficial veins (non-varicose)	1
Alternative diagnosis as likely or wider than that of deep vein thrombosis	−2

*Note.* Low probability: score of 0 or less; Moderate probability: 1–2; High probability: 3 or more.

No prophylactic treatment for DVT was planned. All the patients were monitored for the development of DVT, and treatment was given for symptomatic cases only.

Renal function tests (RFTs) were done post-surgery and at discharge. If the level of serum creatinine was elevated to above 1.4 mg/dL or the level of Blood Urea Nitrogen (BUN) was higher than 25 mg/dL, then elevated RFT level was diagnosed, and medical consultation was ordered.

### Sample size calculation

The sample size was calculated based on a clinically significant effect with at least a 25% reduction in need of blood transfusion in the post-operative period. Assuming the requirement of blood transfusion in control and intervention group to be 15% and 40% respectively [15], with a two-sided significance of 0.05 and a power of 80%, a total of 49 patients in each group would be required.

### Statistical analysis

Statistical analysis was performed using Microsoft Excel 2016. Quantitative data comparison between groups was analyzed using a two-sample student *t*-test. The level of significance was set at *p* < 0.05.

## Results

The demographic details of the 100 patients shown in Table 2 indicate no significant difference between the two groups. Both the groups had a similar number of patients with comorbidities.

**Table 2:** Demographic details of 100 patients.

	Group A	Group B	*p*-Value
Gender	M: 29; F: 21	M: 27; F: 23	0.69
Age (Mean ± SD) (years)	52.7 ± 25.81	49.2 ± 22.22	0.26
Duration of surgery (Mean ± SD) (min)	47.9 ± 11.1	51.8 ± 12.3	0.223
Use of tourniquet in number of patients	27	28	0.842
Comorbidities in number of patients	36	41	0.24

M: Males; F: Females; Group A: TXA group; Group B: Control group.

### Primary outcome measure

The patients receiving TXA (group A) had less need for blood transfusions as seen in Table 3. All the patients whose Hb fell below 9.5 in the post-operative period were given a blood transfusion. Only 7 of the 50 patients needed blood in group A compared to 40 of the 50 patients in group B.

**Table 3:** Comparison of requirement of blood transfusion (post-operative) between the two groups of patients.

Post-op blood transfusion	Group A	Group B
1 unit of blood	7	37
≥2 units of blood	0	3
Total (%)	7 (14%)	40 (80%)
*p*-Value*	*p* < 0.0001	

* Calculated using student *t*-test.

Group A: TXA group; Group B: Control group

### Secondary outcome measure

After surgery, there was a fall in Hb levels in both groups. This fall in Hb was less pronounced in group A, which was statistically significant (*p* < 0.0001). Table 4 illustrates the comparison of Hb levels done in both groups at various times i.e., at the time of admission, one day before surgery, on the day of surgery (after surgery), and at the time of discharge.

**Table 4:** Comparison of Haemoglobin levels done at various times.

Hb levels (Mean ± SD) (range)	Group A	Group B	*p*-Value#
On admission	9.9 ± 0.87 (7.0–11.0)	9.8 ± 0.68 (7.7–10.8)	0.69
Before surgery	10.2 ± 0.46 (9.5–11)	10.1 ± 0.34 (9.6–10.8)	0.15
After surgery	9.8 ± 0.44 (9.0–10.7)	9.2 ± 0.40 (8.5–10.1)	<0.0001*
At discharge	9.8 ± 0.31 (9.5–10.4)	9.9 ± 0.18 (9.6–10.6)	0.21

# Calculated using Student *t*-test (unpaired).

Group A: TXA group; Group B: Control group.

There was a statistically insignificant difference in the length of hospital stay between the two groups. (*p* = 0.6). Among the 100 patients under study, the average length of their hospital stay was six days (ranging from 4 to 10 days) for patients in group A and 7.3 days (ranging from 4 to 11 days) for the patients in group B.

The complications seen in the two groups are summarized in Table 5. There was one mortality in each group. The cause of the mortality was due to poor general condition, decreased cardiac function, and advanced age. Both of these happened within two weeks of the surgery. Both the patients were progressing well when they suddenly deteriorated and collapsed. They could not be revived and were diagnosed with Acute Myocardial Infarction.

**Table 5:** Other complications seen in the two groups.

	Group A	Group B
Death	1	1
Asymptomatic DVT	3	1
Symptomatic DVT	1	0
CVA/stroke	0	0
MI/Acute coronary syndrome	1	1
Deranged renal function tests	4	3

Group A: TXA group; Group B: Control group.

Leg swelling was seen in four patients of group A and one patient of group B. This was confirmed by doing serial measurements of mid-calf circumference. However, none of the patients complained of pain or raised temperature in the calf area. The swelling subsided on its own within two weeks. There were no patients who complained of weakness of any part of the body or other symptoms of cerebrovascular accident (CVA) in either of the groups.

The elevated levels of serum creatinine and/or BUN were seen in four cases of group A, and three cases of group B. Medical consultation was taken for all these cases and managed accordingly.

The safety of using TXA was evaluated by clinical monitoring of side effects and looking for deranged renal function tests. On comparing the complications in both groups, they were found to be similar. The difference in groups A and B was statistically insignificant (*p* = 0.7). Hence, there is no increased risk of using TXA in our patients.

## Discussion

TXA is a lysine analog used frequently as an anti-fibrinolytic agent in trauma and major surgeries [16]. Although TXA in orthopedic surgery has been shown to reduce blood transfusions and decrease mortality in patients, optimal dosing, the timing of administration, mechanism, and pharmacokinetics require further elucidation [7, 16–18]. Recent retrospective studies have not shown evidence of increased thromboembolic events [19].

Our study showed that there was a significant reduction in post-operative blood transfusions. The fall in Hb was less in the TXA group, whereas there was no difference in length of hospital stay in both the groups. Our study also showed no significant rise in complications due to TXA administration.

There were several limitations of our study. The patients were randomized using random number tables without matching for their fracture types and fracture regions. This may result in a probable bias due to different methods of internal fixation and variable duration of surgery, which will, in turn, affect the blood loss.

The dose of TXA used in our study was 1 g, and the timing of administration of TXA to the patient was at the start of the incision. This was as per protocol used by Gausden et al. [20]. Zhang et al. also agreed that the common methods of IV TXA usage are the immediate pre-operative doses of 1 g or 10–15 mg/kg [21]. Lanoiselée et al. pointed out that TXA plasma concentrations were maintained for > 10 mg/L during surgery and for a minimum of 3 h with a pre-operative TXA dose of 1 g in hip arthroplasty [22].

The use of TXA results in reducing the requirement of post-operative blood transfusion in our study. In view of the high risk of infections and anaphylactic reaction due to blood transfusion, the achievement of less blood transfusion by giving TXA is very important [6]. In a meta-analysis by Zhang et al., the most important finding was a reduced transfusion rate due to TXA [21]. Because of this, patients may have an indirect benefit by reduced chance of transfusion-related risks [8, 23, 24]. Moreover, there is a potential financial benefit associated with the use of TXA in hip fracture surgery when considering less requirement of blood transfusion [25]. Due to the reduction in blood transfusion, the resultant cost of procuring and transfusing blood was saved. This would lead to a reduction in the cost of hospitalization. Sassoon et al. [26] also concluded that the use of TXA reduces direct and indirect transfusion-related costs when used in surface replacement arthroplasty. Similarly, Howard et al. [27] found that TXA in patients with postpartum hemorrhage is a cost-effective method to reduce maternal mortality and morbidity.

The difference in fall of Hb levels between the two groups in our study is significant. Zhang et al. in a meta-analysis concluded that IV TXA reduced post-operative hemoglobin decline [21]. Sadeghi et al. also reported a significant difference in post operative hemoglobin level in patients receiving TXA compared to the control group [24].

The complications seen in the two groups of patients are DVT, death, and deranged renal function tests with almost similar incidence. Zhang et al. in their study, also reported no difference in 90-day mortality or rate of thromboembolic events between two groups of patients, one of which received IV TXA and the other being the control group [21]. The study done by Fischer et al. found no difference in mortality or incidence of DVT in patients getting TXA compared to those not getting TXA [28].

## Conclusion

The use of TXA reduces the requirement of blood transfusion in trauma patients, though there is no significant reduction in days of hospital admission. This results in lowering the cost of therapy to the patient. There is no significant rise in complication in the patients who were administered TXA compared to the placebo. This requires further studies on a larger population group to look for the incidence of complications due to TXA.

## Conflict of interest

The authors declare that they have no relevant financial or non-financial interests to report.

## Source of funding

This research did not receive any specific funding.

## Ethical approval

This study received ethical approval from the Institutional Ethics committee (IEC) of Gian Sagar Medical College & Hospital, Banur, Punjab, India, under the protocol number PHMA/GSMCH-17/IEC-29A.

## Informed consent

Written informed consent was obtained from all patients and/or families.

## Author contribution

All the authors (GK, JS, HS, NJD): Conception and design of the work, analysis & interpretation of data & finalized the present version of the manuscript.
